# CDCOCA: A statistical method to define complexity dependence of co-occuring chromosomal aberrations

**DOI:** 10.1186/1755-8794-4-21

**Published:** 2011-03-03

**Authors:** Nitin Kumar, Hubert Rehrauer, Haoyang Cai, Michael Baudis

**Affiliations:** 1 Institute of Molecular Life Sciences, University of Zurich, Winterthurerstrasse 190, Zurich, Switzerland; 2 Functional Genomics Center Zurich, University of Zurich, Winterthurerstrasse 190, Zurich, Switzerland

## Abstract

**Background:**

Copy number alterations (CNA) play a key role in cancer development and progression. Since more than one CNA can be detected in most tumors, frequently co-occurring genetic CNA may point to cooperating cancer related genes. Existing methods for co-occurrence evaluation so far have not considered the overall heterogeneity of CNA per tumor, resulting in a preferential detection of frequent changes with limited specificity for each association due to the high genetic instability of many samples.

**Method:**

We hypothesize that in cancer some linkage-independent CNA may display a non-random co-occurrence, and that these CNA could be of pathogenetic relevance for the respective cancer. We also hypothesize that the statistical relevance of co-occurring CNA may depend on the sample specific CNA complexity. We verify our hypotheses with a simulation based algorithm CDCOCA (complexity dependence of co-occurring chromosomal aberrations).

**Results:**

Application of CDCOCA to example data sets identified co-occurring CNA from low complex background which otherwise went unnoticed. Identification of cancer associated genes in these co-occurring changes can provide insights of cooperative genes involved in oncogenesis.

**Conclusions:**

We have developed a method to detect associations of regional copy number abnormalities in cancer data. Along with finding statistically relevant CNA co-occurrences, our algorithm points towards a generally low specificity for co-occurrence of regional imbalances in CNA rich samples, which may have negative impact on pathway modeling approaches relying on frequent CNA events.

## Background

Genetic alterations are an absolute requirement for malignant neoplasias in humans [1,2]. Both kind of genetic alterations and order of occurrence are important for cancer development and progression [3]. Additionally to sequential event models, large scale analysis of genomes from patient's tumors have shown that multiple genetic abnormalities can promote the development of one cancer entity [4]. Alterations in cancer genome can range from subtle sequence changes (e.g. point mutations) over structural alterations with functional impact on the coding sequence (e.g. generation of fusion genes by chromosomal translocations) to regional or whole-chromosome copy number abnormalities (see e.g. [5-7]).

Through a gene dosage effect, genomic copy number alterations (CNA) may lead to insufficient expression of tumor suppressors or overexpression of proto-oncogenes, respectively. Recurrent CNA have been identified in nearly all cancer entities [8-10]). Comparative Genomic Hybridization (CGH) [11,12] is a genome wide CNA screening technology which has been widely applied throughout the last two decades. Building on the reverse in situ hybridization principle developed for chromosomal CGH [13], genomic microarray technology (aCGH; [14,15]) now utilizes intensity values from up to millions of short DNA sequences to derive regional copy number estimates.

Large data sets from copy number screening experiments should provide a powerful resource for oncogenomic data mining studies. In contrast to expression data, copy number data arises from the projection of discrete values into the experimental space. As such, a reduction of the (a)CGH data can result in the minimal information of segmental status (gain/loss/normal) and genomic position. This facilitates efforts to integrate data across large numbers of experimental series and derived from diverse tumor entities. So far, most of these efforts have been of descriptive nature [10,16] or have been aimed at the definition of disease-specific genomic patterns and useful pattern descriptors ("markers", e.g. [17]). Other publications have attempted the reconstruction of relation and temporal order of oncogenetic events [18-20].

For some cancers types such as subsets of colorectal adenocarcinoma, presence of a limited number of genetic events including several CNA is critical for cancer development [21]. Other neoplasias such as chronic lymphocytic leukemia (CLL) display a paucity of CNA, which however may be correlated to patient survival [22]. These examples illustrate that the presence of certain CNA is not a chance phenomenon, but may either be necessary for cancer development or give a selective edge to affected clones. Previous publications have tried to address the cooperative nature of co-occurring CNA [23,24]. So far, these approaches have not considered the high variability in the complexity of CNAs among individual malignant tumors. Here, we develop an algorithm CDCOCA for analysis of co-occurring oncogenomic CNA events which considers the genomic complexity of the individual samples. We use our approach for detection of CNA events in real-world example data sets. Furthermore, we compare the results from CDCOCA to a previously published method [23] (which we call "analysis 3" in this paper) and also to a modified version of CDCOCA which does not include the adjustment for genomic complexity.

## Methods

### Data

Annotated copy number and associated data was selected from our Progenetix (a)CGH database ([25]: http://www.progenetix.net; status as of 2010-03-01). For model development and testing, we choose one hematopoietic (MCL) and one solid tumor entity (BLCA) due to their overall intermediate genomic complexity, without consideration of their previously established genomic imbalance profiles or CNA subset analysis.

For analysis, copy number status data was determined for 320 genomic intervals based on corresponding cytogenetic bands. Sex chromosomes were removed due to possible bias in some of the published series (e.g. use as normalization control in (a)CGH experiments), resulting in 303 genomic intervals. For analysis by CDCOCA/CICOCA, gain and loss status of all genomic intervals were considered separately, leading to a data matrix with 606 categories. Only genomic intervals showing change in at least one sample were considered for analysis resulting in a CDCOCA/CICOCA input matrix with 593 categories for BLCA and 571 for MCL. For analysis 3, the original data matrix containing 303 genomic intervals was used. As a surrogate score for genomic complexity, a case specific score was calculated by adding each type of genomic imbalances (gain and/or loss) occurring on a chromosomal arm [26].

From now onwards we will use the term "genomic interval" for genomic interval status. A gain and loss association on same chromosome (e.g. -1p and + 1q) will be referred as "bidirectional" change. The modified structure of the data matrices is exemplified in Table 1. Any gain/loss status of a genomic interval is represented by the value 1.

**Table 1:** Binary matrix derived from CGH data.

	g-c1p11	g-c1p12	g-c1p13	l-c1p11	l-c1p12	l-c1p13
1	0	0	1	1	1	0
2	0	0	0	0	0	1
3	0	0	1	1	0	0
4	1	1	1	0	0	0
5	1	1	1	0	0	0
6	0	0	0	0	1	1

For each cytogentic band (e.g. c1p12) occurrence of gain (e.g. g-c1p12) and loss (e.g. l-c1p12) is annotated as separate event for each case.

### Model

Let *D *be the data matrix of dimension *n*x*m*, where n is the number of samples and m is the number of genomic intervals. *D_i,j _*= 1, if a CNA is present in genomic interval j in sample i else *D_i,j _*= 0. *F_j _*represents the number of sample having CNA at genomic interval *j, F_j _*is given by $\sum_{i=1}^{n}D_{ij}$. $P_{w}=(P_{w}^{1}...P_{w}^{n})$ represents the vector of probability weights given to samples. The prior probability weight for any sample *r is *defined by the number of CNAs in patient *r *over total number of CNA across all samples

$$P_{w}^{r}=\frac{\sum_{j=1}^{m}D_{rj}}{\sum_{i=0}^{n}\sum_{j=0}^{m}D_{t,j}}$$

Simulation of any genomic interval *j *is achieved by redistribution of the CNA status over all samples. For genomic interval *j*, we define $D^{*i}=(D_{1}^{*1}...D_{j}^{*n})$ as the corresponding vector representing the CNA status of simulated data. $D_{j}^{*}$ is obtained in a way so that $F_{j}^{*}\approx F_{j}$.

Overlay between two genomic intervals is computed using Jaccard's index [27]. Jaccard's index gives a value between 0 and 1, where one represents a perfect overlap and zero, no overlap. The Jaccard's index between any two genomic intervals j and k is computed as

$$J_{jk}=\frac{N_{jk}^{11}}{N_{jk}^{10}+N_{jk}^{01}+N_{jk}^{11}}$$

$N_{jk}^{11}$ number of samples with CNA in genomic intervals status, j and k.

$N_{jk}^{10}$ number of samples with CNA in genomic interval status j but not k.

$N_{jk}^{01}$ number of samples with CNA in genomic interval status k but not j.

The overlap obtained on permutation is represented by $J_{jk}^{*}$ Frequency of a co-occurrence is computed as

$$F_{jk}=\frac{N_{jk}^{11}}{n}$$

*F_jk _*frequency of an overlap between genomic intervals status i and j.

$N_{jk}^{11}$ number of samples having change in both genomic interval status i and j. *n *total number of samples in the data.

### CDCOCA Algorithm

Let S be the number of simulations and C is the counter measuring the number of times the expected (i.e. permuted) overlap is greater than or equal to the observed overlap. We set the counter of C = 0.

1. Initialize C = 0.

2. Calculate Jaccard's overlap *J_jk _*between genomic interval *j *and *k*.

3. For genomic interval *j *simulate the data to obtain $D_{j}^{*}$ as

a. Obtain a sample index *r *of size 1, from N = (1,....,n) using $P_{w}^{i}$ such that sample with maximum weight given has a higher probability of getting a change on permutation, update $D_{j}^{*r}$ = 1.

b. Update N = N[-r].

c. Update $P_{w}^{i}=P_{w}^{i}[-r],\;P_{w}^{i}=\frac{P_{w}^{i}}{\sum_{i}P_{w}^{i}},\;P_{w}^{i}=\frac{P_{w}^{i}}{1=P_{w}^{i}}$.

d. Repeat step 3a and 3b *F_j _*times to obtain simulated vector $D_{j}^{*}$.

4. For genomic interval *k *simulate the data using step 3 to obtain $D_{k}^{*}$.

5. Recompute Jaccard's overlap $J_{jk}^{*},\;\text{if}\;J_{jk}^{*}\geq \;j_{jk}$ increase *C *= *C *+ 1.

6. Repeat step 3, 4 and 5 for S times.

7. At the end of S (5000 in our case) permutations calculate *p *value as, $p=\frac{C}{S}$.

The *p*-value obtained after step 7 represent the probability of co-occurrence of two CNAs in absence of any other CNA in sample. A low *p*-value cut off will help in enriching for CNAs which occur together even in less heterogenous samples.

## Results and Discussion

We here propose a methodology named CDCOCA (Complexity dependence of co-occurring chromosomal aberrations) that defines highly correlated pairs of CNA in cancer samples while correcting for the overall degree of genomic instability.

**Figure 1:**
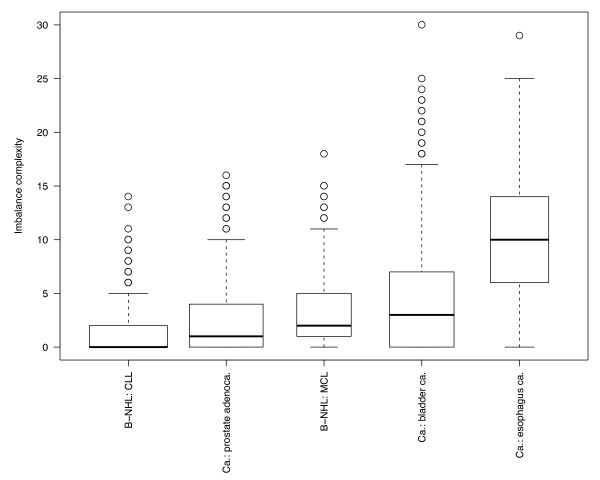
**Complexity boxplot of CNA in some selected cancers**. Box plot for the overall CNA complexity in selected cancer entities. As a surrogate marker for genomic complexity, each cytogenetic arm was scored independently for gains and losses (i.e., max. score of 4 for a chromosome with both gains and losses on both arms), and chromosome scores were summarized for each case.

We determine CNA complexity based on the number of segmental CNA in a sample while accounting for variations introduced through different resolutions and/or segmentation algorithms [10]. A sample is called "CNA complex" if it has acquired a high number of CNA, and conversely "CNA simple" if a low number of segmental imbalances have been detected. In Figure 1 the distribution of copy number complexities is presented for data from selected tumor entities, extracted from the Progenetix database.

The performance of CDCOCA depends on the number of tumor samples, number of genomic intervals and number of iterations. CDCOCA produces a matrix of p values for all possible associations in the data matrix which are then used to enrich for associations dependent on sample complexity. The algorithm is implemented in the R statistical framework and is available through R package "CDCOCA" provided on the Progenetix website [25].

**Figure 2:**
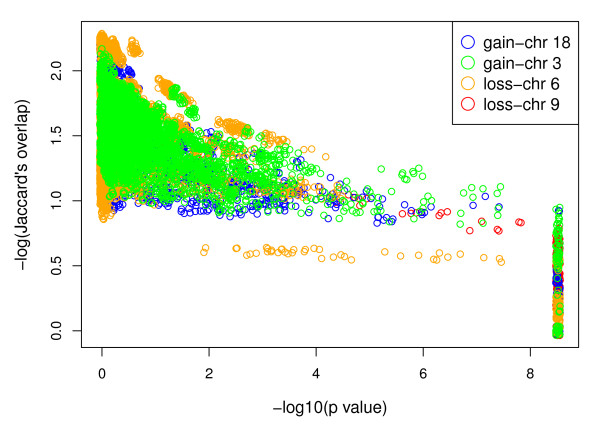
**Log of p value plotted against log of Jaccard's index for BLCA**. For simplicity reasons all the associations involving only 4 chromosomal changes are shown here. Each color dot reprent an association of that particular chromosome with some other chromosomal band. Most of the associations have a low Jaccard's index and very high p values (upper left side of plot) these associations represent CNA in CNA complex samples. Few associations have a high Jaccard's index and low p values (lower right side of plot); these associations are present in "CNA simple" samples.

**Figure 3:**
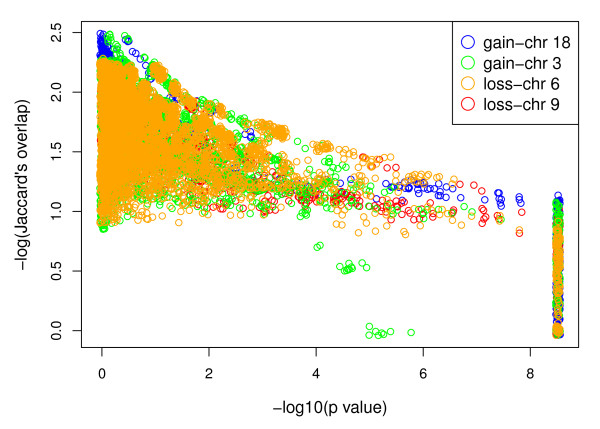
**Log of p value plotted against log of Jaccard's index for MCL**. Most of the associations have a very high CNA complex background (upper right side of plot) whereas a associations with high Jaccard's index and low p value (lower right side) are also present for all chromosomes.

We applied the CDCOCA algorithm to bladder carcinoma (BLCA) and mantle cell lymphoma (MCL) copy number data, considering gains and losses for each interval as separate events. The readout of the analyses consisted of the p values obtained after randomization for all observed associations in both cancers after 5000 permutations each. We used Jaccard's index to calculate the overlap between genomic intervals [27]. Figure 2 and 3 show the log of p values plotted against the log of Jaccard's index. For simplicity, here p values for only 4 chromosomal changes were plotted. Using CDCOCA we observed that most of the genetic associations have very low Jaccard's overlap and arise from genetic changes which occur in CNA complex samples (hence high p values). Associations presenting with high Jaccard's indices and low p-values represent CNA with high probability of specific co-occurrence (i.e. frequent co-occurrence independent of high sample CNA complexity).

Our results show that most of the CNA data for both cancers are derived on a background of multiple and extended CNA. The total number of genetic associations in both cancer types remains beyond scope of the current analysis. However, with CDCOCA we are able to focus on a defined set of statistically relevant, specific changes.

For estimating the performance of our methodology in relation to otherwise discussed models we compared CDCOCA to a modified version "CICOCA" (see supplement) and a previously published method [23]. Both the later algorithms do not include an estimate of sample complexity and primarily identify associations with a high frequency. CICOCA and analysis 3 use different methods to compute overlap resulting in slightly different but overall concordant results.

**Figure 4:**
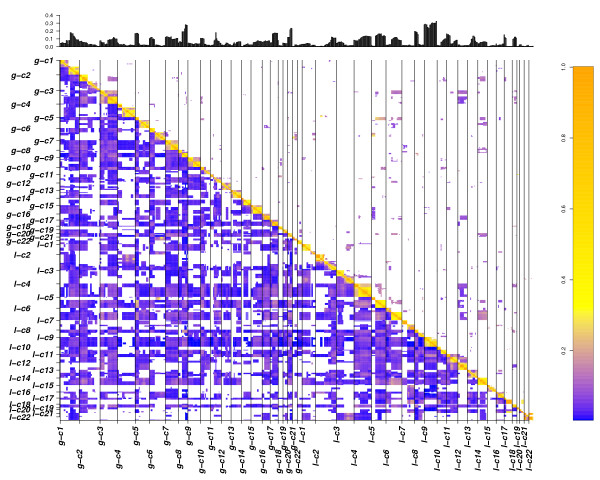
**Matrix plot showing results obtained for BLCA with CDCOCA**. The diagonal lower half of the image represents all the possible associations in BLCA data where as diagonal upper half represents associations obtained after p value cut off. Frequency of genomic intervals is represented by histogram at the top. Color code represents the value of Jaccard's overlap between associations. The high correlation throughout the diagonal confirms shows the strong connection between CNA co-occurrence and close genetic linkage.

**Figure 5:**
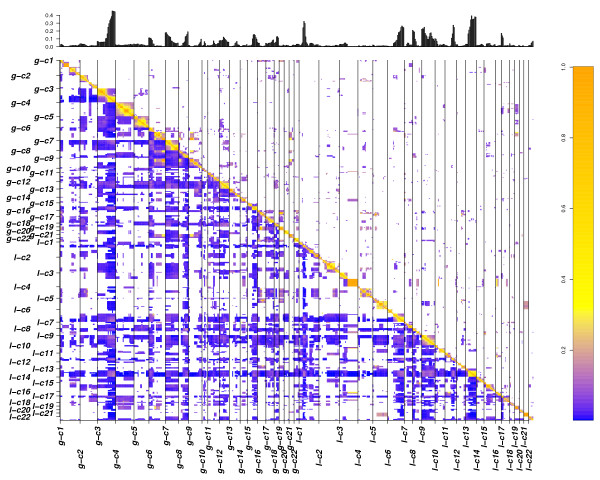
**Matrix plot showing results obtained for MCL with CDCOCA**. The diagonal lower half of the image represents all the possible associations in BLCA data where as diagonal upper half represents associations obtained after p value cut off. Frequency of genomic intervals is represented by histogram at the top. Color code represents the value of Jaccard's overlap between associations. The high correlation throughout the diagonal confirms shows the strong connection between CNA co-occurrence and close genetic linkage.

With CICOCA, a high number of co-occurring changes were obtained after p value cut off (Figure 1 and 2 in additional file 1). In contrast, introduction of complexity estimation leads to a focus on changes arising on a low complexity background (Figure 4 and 5). With analysis 3 (Figure 3 and 4 in additional file 1) a very low number of associations was obtained in our sample data set. As expected these only involved high frequent changes. We could show that most of the CNA obtained by analysis 3 (Figure 3 and 4 in additional file 1) were also detected using CDCOCA (Figure 4 and 5) and CICOCA (Figure 1 and 2 in additional file 1). CICOCA and analysis 3 can be used to describe frequent associations, while CDCOCA additionally allows to test the specificity of associations and to apply thresholds accordingly. Compared to frequency based thresholding, one advantage of CDCOCA is its independence from arbitrary cut-off values. The algorithm scores every association. The p value obtained assigns a statistical significance to the associations which is independent of the frequency of the association in the data but takes the complexity of the sample into account.

### Bladder carcinoma

An overview of the most frequent genomic imbalances in urinary BLCA can be found in e.g. [10]. Most frequent gains in BLCA include regions on 1q, 5p, 8q,17, 19 and 21q, while the most frequent losses occur on 2q, 4, 5q, 6q, 8p, 9, and 13q (Figure 1 and 3 in additional file 1 and Figure 4 barplot). Due to the high degree of aneuploidy in BLCA, CNA data is highly complex (Figure 4 matrix plot) resulting in a very high number of total associations (Table 2).

**Table 2:** Statistic of associations in BLCA

	Analysis method	Total associations	No. intra-chromosomal associations	p-value	FDR	Associations obtained	No. intera-chromosomal associations
1	CDCOCA	96436	4786	0.02	0.275	6991	3619

2	CICOCA	96436	4786	0.02	0.096	20089	3891

2	Analysis 3	40284	2577	0.02	0.7211	321	152

**Figure 6:**
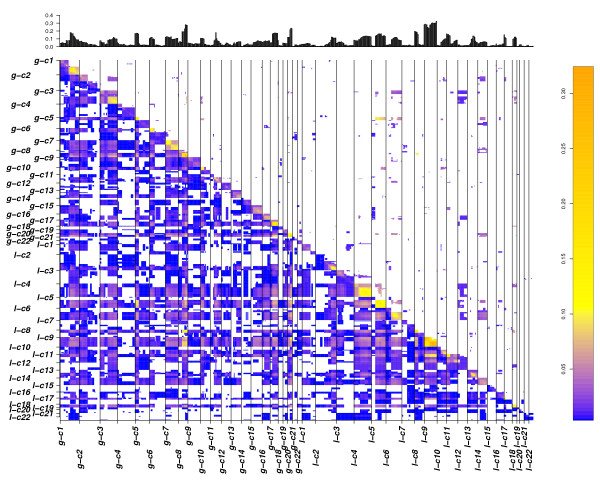
**Matrix plot showing frequency of associations in BLCA**. Associations are shown similar to figure 4 but here displaying frequencies of associations in place of Jaccard's overlap values. Most frequent CNA associations involve genetically linked CNA. Associations involving high frequent changes are lost after p value cut off indicating their occurrence in a CNA complex background.

A large proportion of associations combine a low frequency with a high Jaccard's index (Figure 4 and 6 matrix plots). We applied a p-value cut off of 0.02 resulting in a false discovery rate (FDR) of 27.5%. At this p-value cut off, 75% of intra-chromosomal associations passed the threshold, confirming the correlation between genetic linkage and involvement in CNA events. Table 2 contains the information about the comparison of results for all three analysis. For simplicity reasons here we limit the display to the 100 most frequent inter-chromosomal changes obtained after p-value cut off.

**Figure 7:**
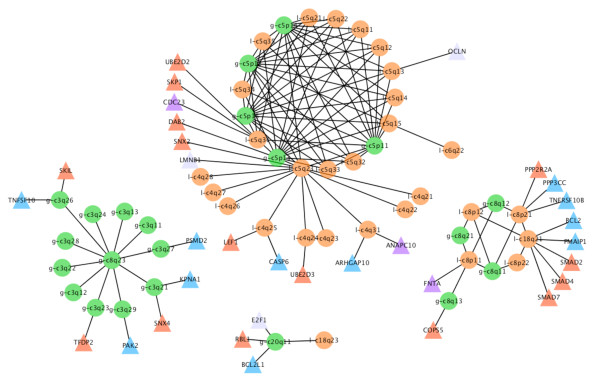
**100 frequent associations obtained after p value cut off in BLCA with CDCOCA**. Pathway associations for presumptive target genes from the 100 most frequent associations in BLCA obtained after p value cut-off are shown. TGF-beta signaling genes (blue triangles) and genes from cellular apoptotic pathways (red triangles) present in these genetic locations are displayed. Gains are represented with prefix "g" and green color circles whereas losses with prefix "l" and orange circles. Associations were drawn using cytoscape [42].

According to CDCOCA, specific pairs of genomic imbalances in bladder carcinoma include concurrent "bidirectional" losses on 8p and gains on 8q (Figure 7). In the comparative analysis, gains involving chromosome 8q were detected with all three methods (Figure 5 and 6 in additional file 1 and Figure 7). However, with CDCOCA the frequent co-occurrence of these CNA on the background of a low genomic complexity became more apparent. This observation may point to an early appearance of these CNA during tumorigenesis, with a possible role as cancer initiating event. While gains on distal 8q are the most consistent copy number change in epithelial neoplasias with MYC considered a predominant target, recently deletions on 8p23.3 have been associated with aggressive clinical behavior in BLCA [28]. Another observation concerned changes involving concurrent gains on 5p and losses on 5q which were also associated with losses on chromosome 4q and distal 6q (6q22). These co-occurrences (Figure 5 and 6 in additional file 1 and Figure 7). Although one may assume that "bidirectional" changes involving both chromosomal arms are based on simple cytogenetic events, e.g. isochromosome formation, the limitation of this pattern to distinct chromosomes points at an evolutionary advantage of both gain and loss accumulation for the malignant clone. Other event pairs obtained by CDCCOA include gains on 8q23 along with gains on 3q, as well as gain on 20q11 with loss on 18q23.

The abundance of 8p losses, 8q gains, 5q losses, 5p gains, 3q gains, 4q losses points towards the importance of these CNA in tumors carrying them. Genes from TGF-beta receptor signaling (blue triangles) and cellular apoptotic pathways (red triangles) located to the co-occurring changes are shown in Figure 7. The presence of genes from the same pathways on co-occurring CNA point towards a possible cooperative action of these genes. CDC23 (5q31), CASP6 (4q25) and PMAIP1 (18q21) are among TGF-receptor cascade genes with well established role in cancer [29,30] Other possible targets for genetic cooperation include PMSD2, PAK2, BCL2L1 and FNTA. Genes from apoptotic signaling pathways mapped to these regions include CDC23 (5q31), SMAD2 (18q21), SMAD4 (18q21) and SMAD7 (18q21) which have been shown defective in several cancer entities [31]. As possible target on 5p, loss of SKP2 had been shown to cause cell senescence [32]. On 5q, loss of function mutations including copy number losses of both APC and MCC have been associated with a variety of epithelial neoplasias [33-36].

### Mantle cell lymphoma

**Figure 8:**
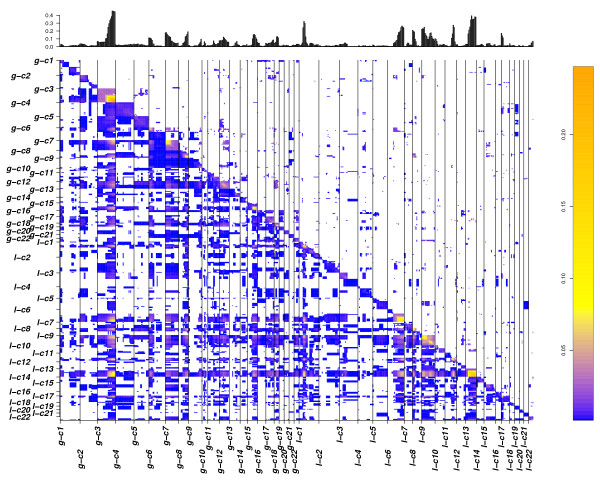
**Matrix plot showing frequency of associations in MCL**. Associations are shown similar to figure 7 but here displaying frequencies of associations in place of Jaccard's overlap values. Most frequent CNA associations involve genetically linked CNA. Associations involving high frequent changes are lost after p value cut off indicating their occurrence in a CNA complex background.

For MCL, an overall p value distribution similar to that of BLCA was observed (Figure 3). Most common CNA in MCL included gains on chromosomes 3q, 6p, 7p and 8q, while most common losses involved regions on 6q, 8p, 9, 11q and 13q (Figure 7 and 8 in additional file 1 and Figure 5).

**Figure 9:**
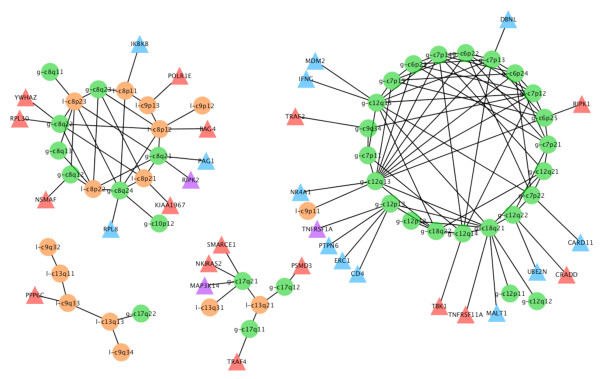
**100 frequent associations obtained after p value cut off in MCL with CDCOCA**. Pathway associations for presumptive target genes from the 100 most frequent associations in MCL. TNF-alpha signaling genes (red triangles) and T-cell receptor signaling pathway genes (blue triangles) present in these genetic locations are shown. Gains are represented with prefix "g" and green color circles whereas losses with prefix "l" and orange circles.

A p value cut-off of 0.04 giving a FDR of 30% was applied with CDCOCA (Table 3 and Figure 8). About 80% of intra-chromosomal associations passed this threshold, representing approx. 50% of all post cut-off associations. The 100 strongest associations obtained with CDCOCA are shown in Figure 9. As in BLCA, CDCOCA detected losses on 8p with gains on 8q, which was not described as association in the other analyses. Also, only CDCOCA selected groups of co-occurrences involving low frequency CNA (e.g. associations involving gains 7p, 6p, 12p and 18q). Other changes such as losses on highly occurring 13q along with gains on not so frequently occurring 7q were obtained using CDCOCA and not with CICOCA and analysis 3 in the top 100 events (Figure 7 and 8 in additional file 1).

**Table 3:** Statistic of associations in MCL

	Analysis method	Total associations	No. intra-chromosomal associations	p-value	FDR	Associations obtained	No. intera-chromosomal associations
1	CDCOCA	57644	3918	0.04	0.30	7513	3175

2	CICOCA	57644	3918	0.04	0.197	11673	3918

2	Analysis 3	31136	2418	0.04	0.571	867	207

As candidate targets, TNF-signaling genes (red triangles) and T-cell receptor signaling genes (blue triangles) are marked on their corresponding band locations in Figure 9. The role of genes such as MDM2 (12p15), TNFRSF1A (12p13), MALT1 (18q21) for neoplastic transformation and/or progression has already been well established [37-39]. Other examples for cancer relevant genes mapping to those regions are STAT2 (12q13), and STAT3 (17q) [40,41].

## Conclusions

We have developed a method CDCOCA to define complexity dependence of co-occurring CNA in cancer samples. In contrast to methods published previously [23] and a modified algorithm which does not include the complexity adjustment step, CDCOCA does not simply focus on the most frequent co-occurrences of regional genomic copy number changes in cancer entities. Here, we determine statistically relevant co-occurring CNA through accounting for the CNA "background noise", introduced e.g. through chromosome scale imbalances (e.g. isochromosomes, chromosomal aneuploidy). In theory, this procedure should highlight specific but comparatively rare CNA events.

As indicated by our analysis of BLCA and MCL, two unrelated cancer entities with overall intermediate copy number complexity, the relevant CNA associations in many specimen are obscured due to the large number and/or extension of regional CNA. When correcting for genomic background heterogeneity most of the associations involving highly recurring CNA were removed. This indicates that many high frequency changes may be related to the overall genomic instability and therefore cannot unanimously be assigned a causative role in oncogenesis. Especially regarding the large number of genes affected by complex genomic imbalances, some of the cancer type specific CNA patterns may represent an epiphenomenon of disturbed genomic maintenance processes rather than the expression of copy number dependent target gene modifications.

However, when accounting for the overall complexity, CNA associations may point towards connected events and/or preferred pathways activated during carcinogenesis. Based on our CNA associations, we found multiple genes from single well defined cancer pathways to be a effected in sample subsets. Alteration of more than one gene in a pathway may potentiate the effect on pathway function and be responsible for a specific clonal phenotype.

CDCOCA should prove to be a powerful tool for defining mutual associations at gene level and to gain insights into cellular mechanisms relevant for oncogenesis. Although we applied our method to CGH data at band resolution, there is no practical obstacle against use with segmented data from high resolution genomic array experiments. In fact, this should facilitate a gene centric analysis and automatic integration with functional data sources.

## Competing interests

The authors declare that they have no competing interests.

## Authors' contributions

NK, MB, HR designed and conceived the experiments; NK implemented the software; NK, MB analyzed the data, HC, MB contributed to the data. All authors read and approved the final manuscript.

## Pre-publication history

The pre-publication history for this paper can be accessed here:

http://www.biomedcentral.com/1755-8794/4/21/prepub

## Supplementary Material

Additional file 1 **CICOCA: A method to define complexity independence of co-occurring chromosomal aberrations**. The additional file contains information about the statistical method CICOCA which is compared with CDCOCA. This method (as described in text above) aims in finding co-occurring chromosomal associations independent of the sample complexity. In addition to CICOCA this file also contains all the additional figures which are referred in the paper along with a detail description of all the additional figures. Click here for file
