# Supplementary material for: CDCOCA: A statistical method to define complexity dependence of co-occuring chromosomal aberrations
Source: BMC Med Genomics. 2011 Mar 3;4:21. doi: 10.1186/1755-8794-4-21 (PMC3061884; doi:10.1186/1755-8794-4-21)
Supplement: Additional file 1 — CICOCA: A method to define complexity independence of co-occurring chromosomal aberrations. The additional file contains information about the statistical method CICOCA which is compared with CDCOCA. This method (as described in text above) aims in finding co-occurring chromosomal associations independent of the sample complexity. In addition to CICOCA this file also contains all the additional figures which are referred in the paper along with a detail description of all the additional figures. [file 1755-8794-4-21-S1.PDF]

Additional file to

CDCOCA: A statistical method to define complexity dependence of co-occuring chromosomal aberrations

Nitin Kumar[1], Hubert Rehrauer[2] , Haoyang Cai [1] and Michael Baudis*[1]

[1]Institute of Molecular Life Sciences, University of Zurich, Winterthurerstrasse 190, Zurich, Switzerland,
[2]Functional Genomics Center Zurich, University of Zurich, Winterthurerstrasse 190, Zurich, Switzerland

Email: Nitin Kumar - nitin.kumar@imls.uzh.ch; Hubert Rehrauer - hubert.rehrauer@fgcz.ethz.ch; Haoyang Cai - haoyang.cai@imls.uzh.ch; Michael Baudis *- michael.baudis@imls.uzh.ch;

*Corresponding author

Methods used to compare with CDCOCA

CICOCA Algorithm

Let S be the number of simulations and C the counter measuring the number of times the expected (i.e. simulated) overlap is greater than or equal to the observed overlap. We set the counter of C = 0.

1. Initialize C = 0.
2. Calculate Jaccard's overlap J_{jk} between genomic interval j and k.
3. For genomic interval j shuffle all the values in the column keeping column sum constant to obtain D_j^*.
4. For genomic interval k simulate the data using step 3 to obtain D_k^*.
5. Recompute Jaccard's overlap J_{jk}^*, if $J_{jk}^* \geq J_{jk}$, increase $C = C + 1$.
6. Repeat step 3, 4 and 5 for S times.
7. At the end of S (5000 in our case) times calculate p value as, $p = \frac{C}{S}$.

The p-value obtained after step 7 represent the probability of co-occurrence of two CNAs. A low p-value cut off will help in enriching for CNAs which occur together more often than by chance.

Analysis 3 using Bredel et. al, 2009 (represented as analysis 3))was performed. The overlap for analysis 3 is computed as

$$S_{jk} = \frac{\sum_{i=1}^{n} D_{ij} * D_{ik}}{n_{jk}}$$

$D_{ij} * D_{ik}$ scores +1 if cytobands j,k for tumor i are gained or lost together and -1 if one is gained and other is lost and 0 when both have no change or one has a change.

n_{jk} is the number of tumors with both j and k altered.

Figure 1 - Matrix plot showing results obtained for BLCA with CICOCA

The diagonal lower half of the image represents all the possible associations in BLCA data where as diagonal upper half represents associations obtained after p value cut off using CICOCA. Frequency of genomic intervals is represented by histogram at the top. Color code represents the value of Jaccard's overlap between associations. The high correlation throughout the diagonal confirms shows the strong connection between CNA co-occurrence and close genetic linkage.

Figure 2 - Matrix plot showing results obtained for MCL with CICOCA

The diagonal lower half of the image represents all the possible associations in BLCA data where as diagonal upper half represents associations obtained after p value cut off using CICOCA. Frequency of genomic intervals is represented by histogram at the top. Color code represents the value of Jaccard's overlap between associations. The high correlation throughout the diagonal confirms shows the strong connection between CNA co-occurrence and close genetic linkage.

Figure 3 - Matrix plot showing results obtained for BLCA with analysis 3

The diagonal lower half of the image represents all the possible associations in BLCA data where as diagonal upper half represents associations obtained after p value cut off using analysis 3. Frequency of change of genomic intervals is represented by histogram at the top. Color code represents the value of overlap coefficient between associations.

Figure 4 - Matrix plot showing results obtained for MCL with analysis 3

The diagonal lower half of the image represents all the possible associations in BLCA data where as diagonal upper half represents associations obtained after p value cut off using analysis 3. Frequency of change of genomic intervals is represented by histogram at the top. Color code represents the value of overlap coefficient between associations.

Figure 5 - 100 frequent associations obtained after p value cut off for analysis of BLCA with CICOCA

100 frequent associations obtained after p value cut off for analysis of BLCA with CICOCA are shown. Gains are represented with prefix "g" and green color circles whereas losses with prefix "l" and orange circles.

Figure 6 - 100 frequent associations obtained after p value cut off for analysis of BLCA with analysis 3

100 frequent associations obtained after p value cut off for analysis of BLCA with analysis 3 are shown. Associations of in with both the bands are either gained or lost are represented with green connections whereas associations in which one band is gained and other is lost are represented by orange connections.

Figure 7 - 100 frequent associations obtained after p value cut off for analysis of MCL with CICOCA

100 frequent associations obtained after p value cut off for analysis of BLCA with CICOCA are shown. Gains are represented with prefix "g" and green color circles whereas losses with prefix "l" and orange circles.

Figure 8 - 100 frequent associations obtained after p value cut off for analysis of MCL with analysis 3

100 frequent associations obtained after p value cut off for analysis of BLCA with analysis 3 are shown. Associations of in with both the bands are either gained or lost are represented with green connections whereas associations in which one band is gained and other is lost are represented by orange connections.

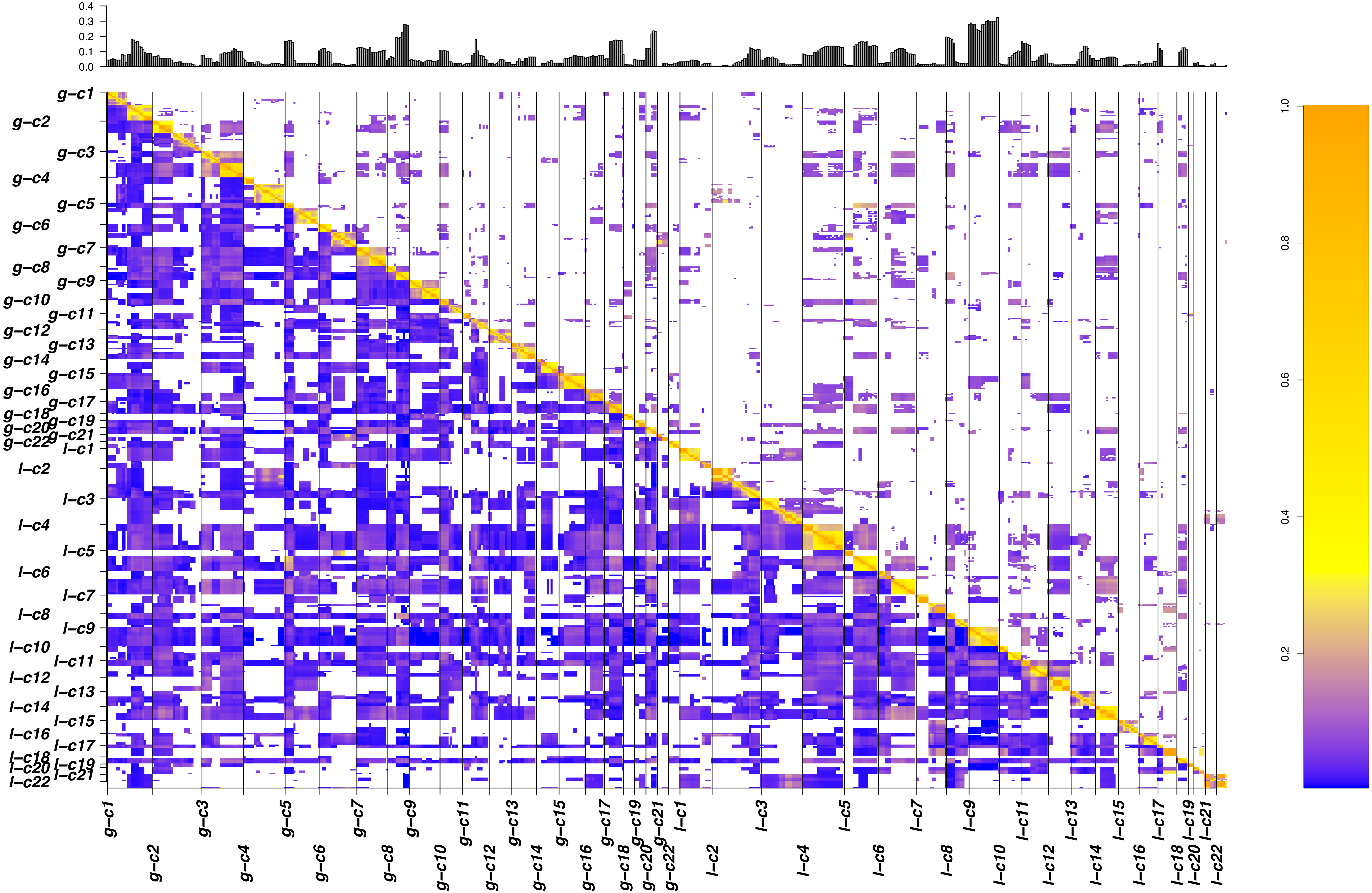

0.4
0.3
0.2
0.1
0.0
g−c1
g−c2
g−c3
g−c4
g−c5
g−c6
g−c7
g−c8
g−c9
g−c10
g−c11
g−c12
g−c13
g−c14
g−c15
g−c16
g−c17
g−c18
g−c19
g−c20
g−c21
g−c22
l−c1
l−c2
l−c3
l−c4
l−c5
l−c6
l−c7
l−c8
l−c9
l−c10
l−c11
l−c12
l−c13
l−c14
l−c15
l−c16
l−c17
l−c18
l−c19
l−c20
l−c21
l−c22
1.0
0.8
0.6
0.4
0.2

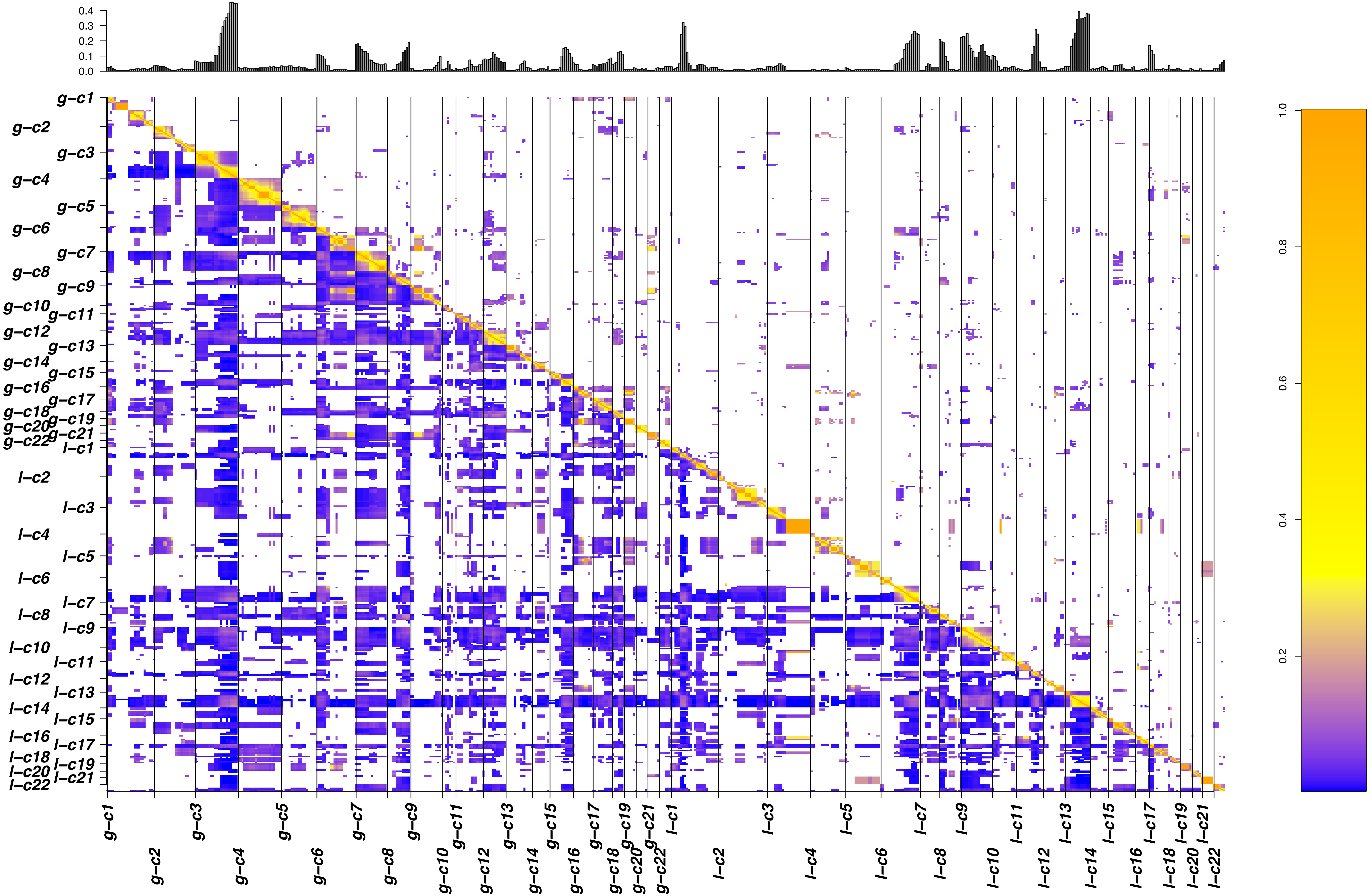
0.4
0.3
0.2
0.1
0.0
g−c1
g−c2
g−c3
g−c4
g−c5
g−c6
g−c7
g−c8
g−c9
g−c10
g−c11
g−c12
g−c13
g−c14
g−c15
g−c16
g−c17
g−c18
g−c19
g−c20
g−c21
g−c22
l−c1
l−c2
l−c3
l−c4
l−c5
l−c6
l−c7
l−c8
l−c9
l−c10
l−c11
l−c12
l−c13
l−c14
l−c15
l−c16
l−c17
l−c18
l−c19
l−c20
l−c21
l−c22
1.0
0.8
0.6
0.4
0.2

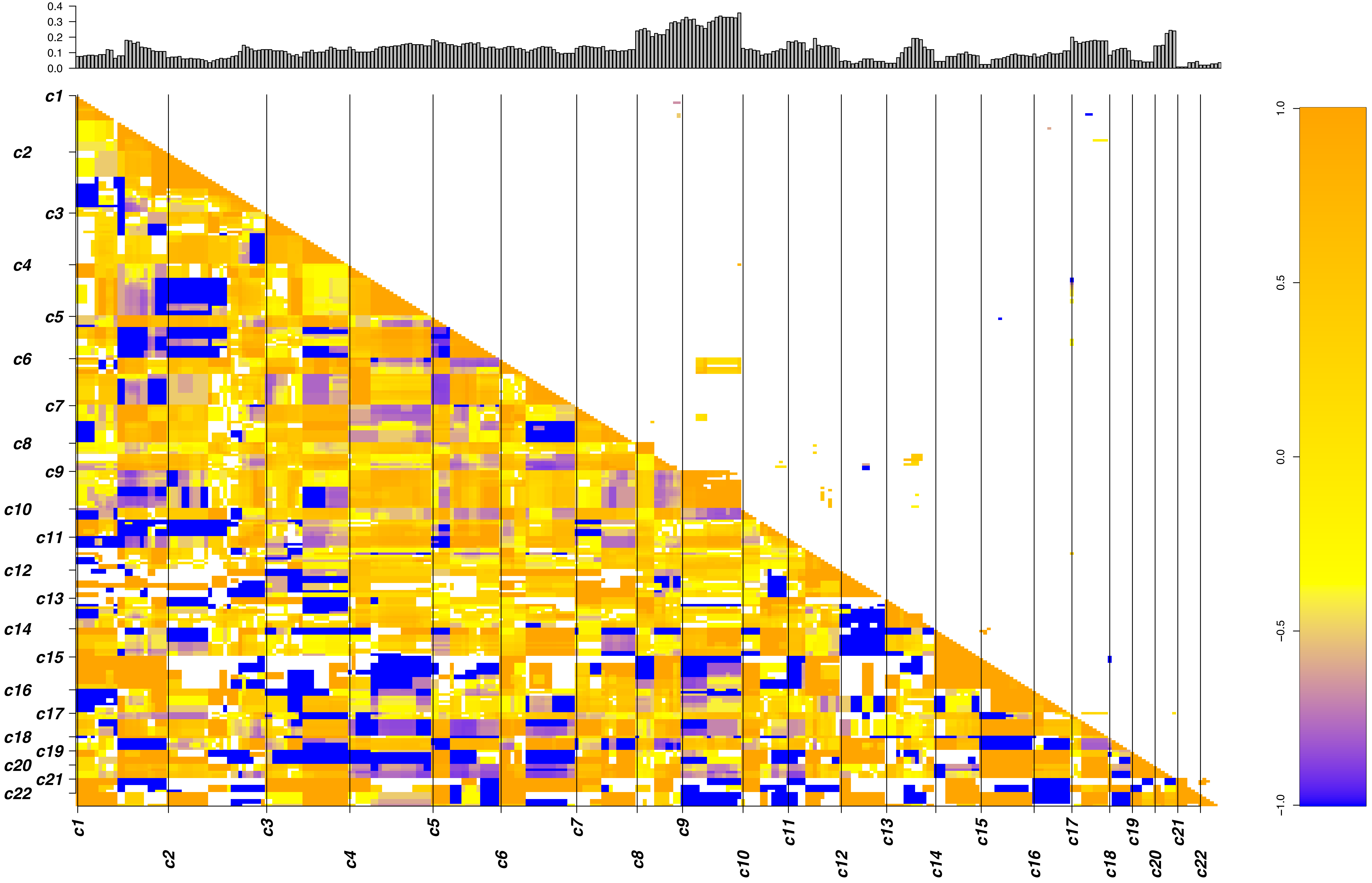
0.4
0.3
0.2
0.1
0.0
c1
c2
c3
c4
c5
c6
c7
c8
c9
c10
c11
c12
c13
c14
c15
c16
c17
c18
c19
c20
c21
c22
1.0
0.5
0.0
−0.5
−1.0

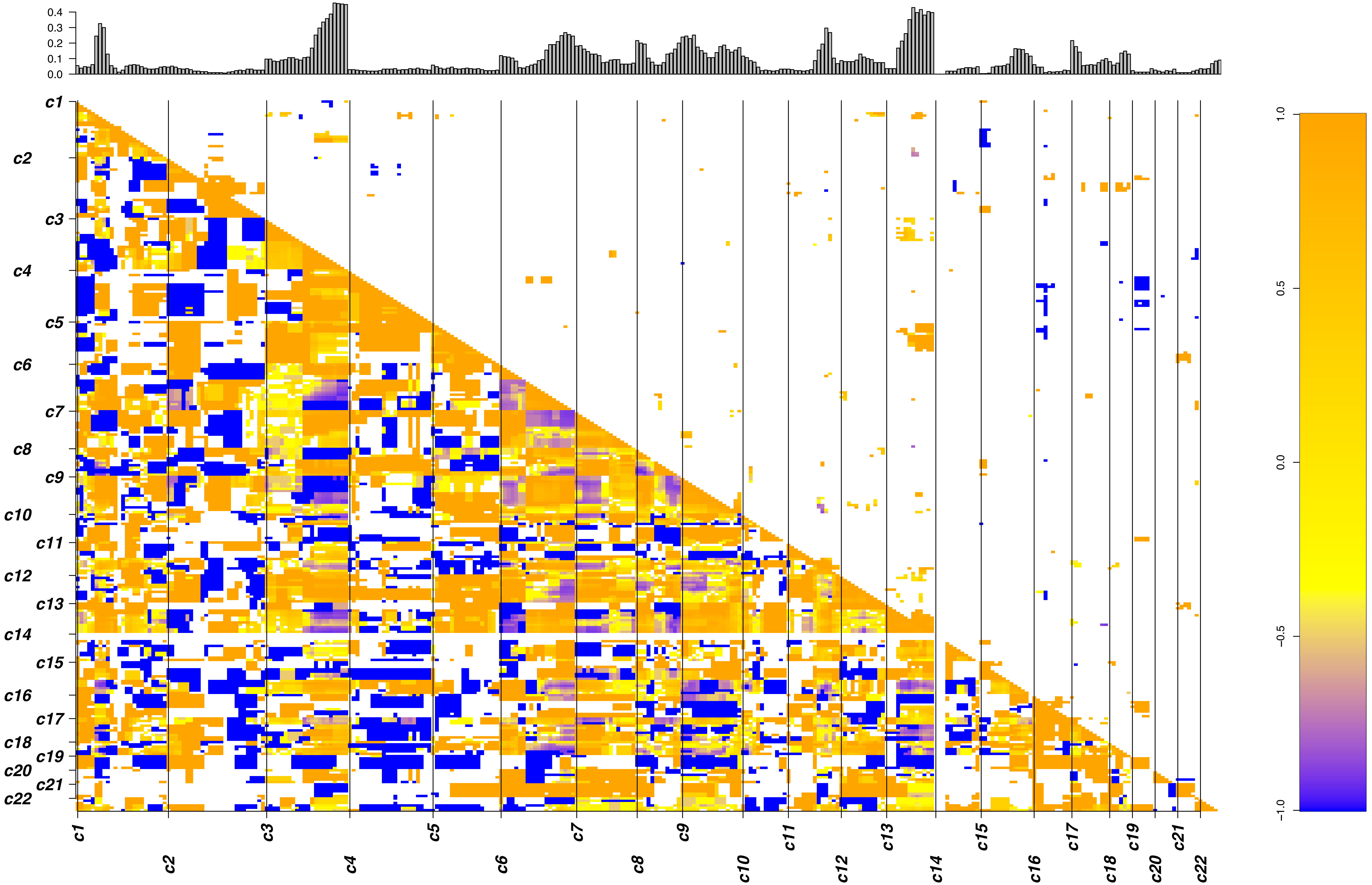

0.4
0.3
0.2
0.1
0.0
c1
c2
c3
c4
c5
c6
c7
c8
c9
c10
c11
c12
c13
c14
c15
c16
c17
c18
c19
c20
c21
c22
1.0
0.5
0.0
−0.5
−1.0

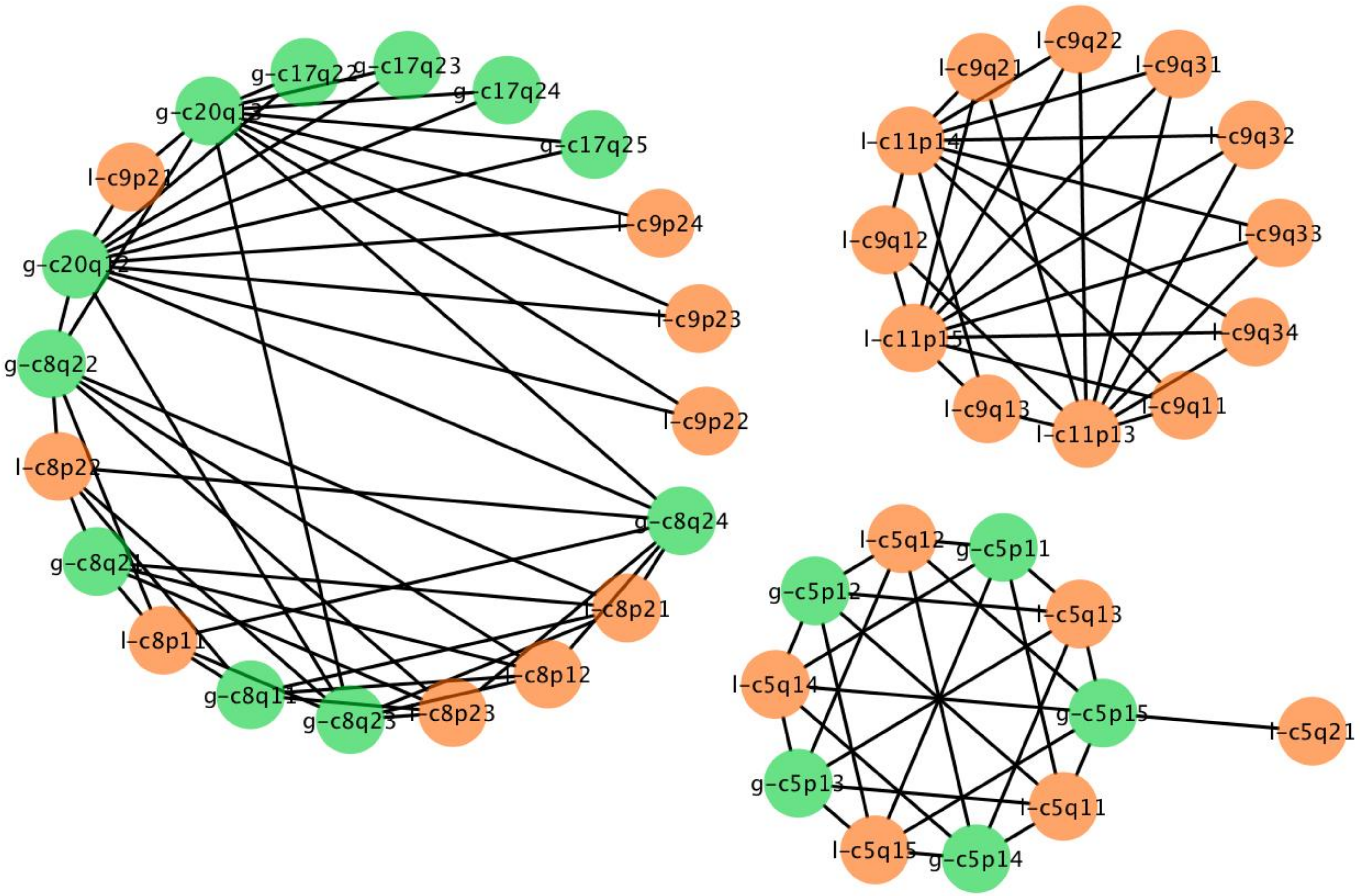
g-c17q22
g-c17q23
g-c17q24
g-c20q13
g-c17q25
l-c9p21
l-c9p24
g-c20q12
l-c9p23
g-c8q22
l-c9p22
l-c8p22
g-c8q24
g-c8q21
l-c8p21
l-c8p11
l-c8p12
g-c8q11
g-c8q23
l-c8p23
l-c9q22
l-c9q21
l-c9q31
l-c11p14
l-c9q32
l-c9q12
l-c9q33
l-c11p15
l-c9q34
l-c9q13
l-c9q11
l-c11p13
l-c5q12
g-c5p11
g-c5p12
l-c5q13
l-c5q14
g-c5p15
l-c5q21
g-c5p13
l-c5q11
l-c5q15
g-c5p14

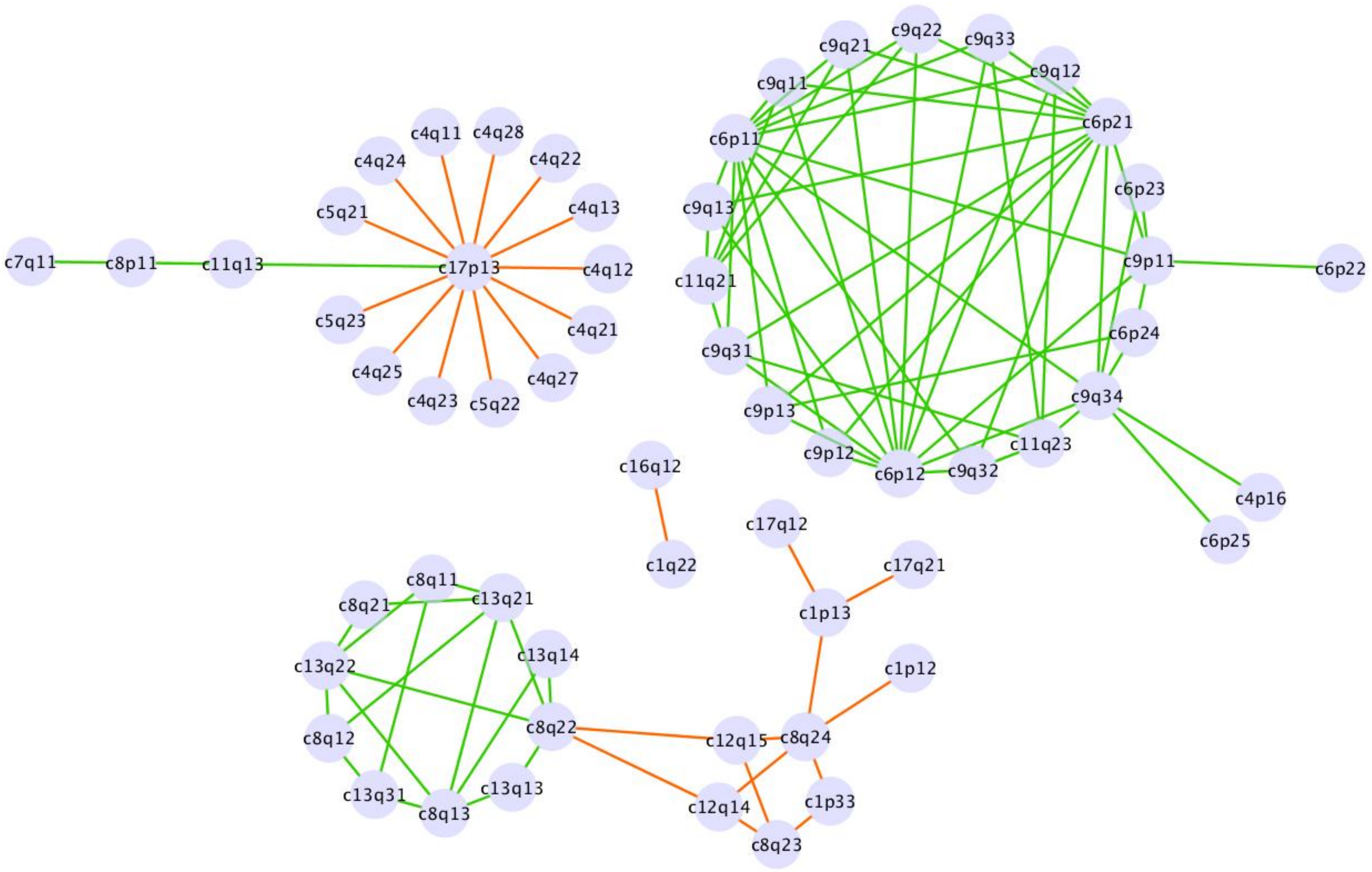
c7q11
c8p11
c11q13
c17p13
c4q24
c4q11
c4q28
c4q22
c5q21
c4q13
c4q12
c5q23
c4q21
c4q25
c4q27
c4q23
c5q22
c9q21
c9q22
c9q33
c9q12
c9q11
c6p21
c6p11
c6p23
c9q13
c9p11
c6p22
c11q21
c6p24
c9q31
c9q34
c9p13
c11q23
c9p12
c6p12
c9q32
c4p16
c6p25
c16q12
c1q22
c17q12
c17q21
c1p13
c8q11
c8q21
c13q21
c13q14
c13q22
c1p12
c8q22
c8q12
c12q15
c8q24
c13q13
c13q31
c8q13
c12q14
c1p33
c8q23

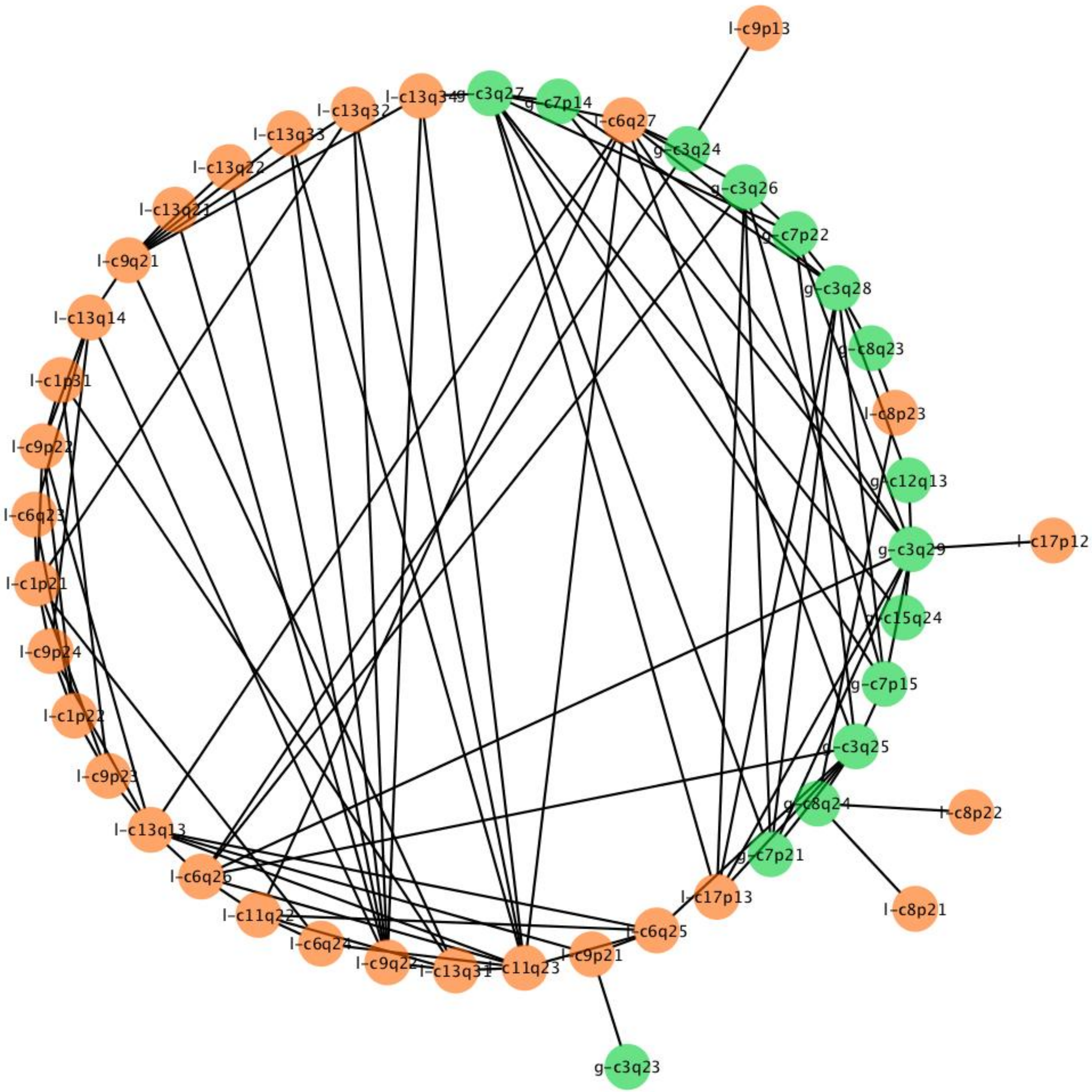
l−c9p13
l−c13q34
g−c3q27
g−c7p14
l−c13q32
l−c6q27
l−c13q33
g−c3q24
l−c13q22
g−c3q26
l−c13q21
g−c7p22
l−c9q21
g−c3q28
l−c13q14
g−c8q23
l−c1p31
l−c8p23
l−c9p22
g−c12q13
l−c6q23
g−c3q29
l−c17p12
l−c1p21
g−c15q24
l−c9p24
g−c7p15
l−c1p22
g−c3q25
l−c9p23
g−c8q24
l−c8p22
l−c13q13
g−c7p21
l−c6q26
l−c17p13
l−c8p21
l−c11q22
l−c6q25
l−c6q24
l−c9q22
l−c13q31
l−c11q23
l−c9p21
g−c3q23

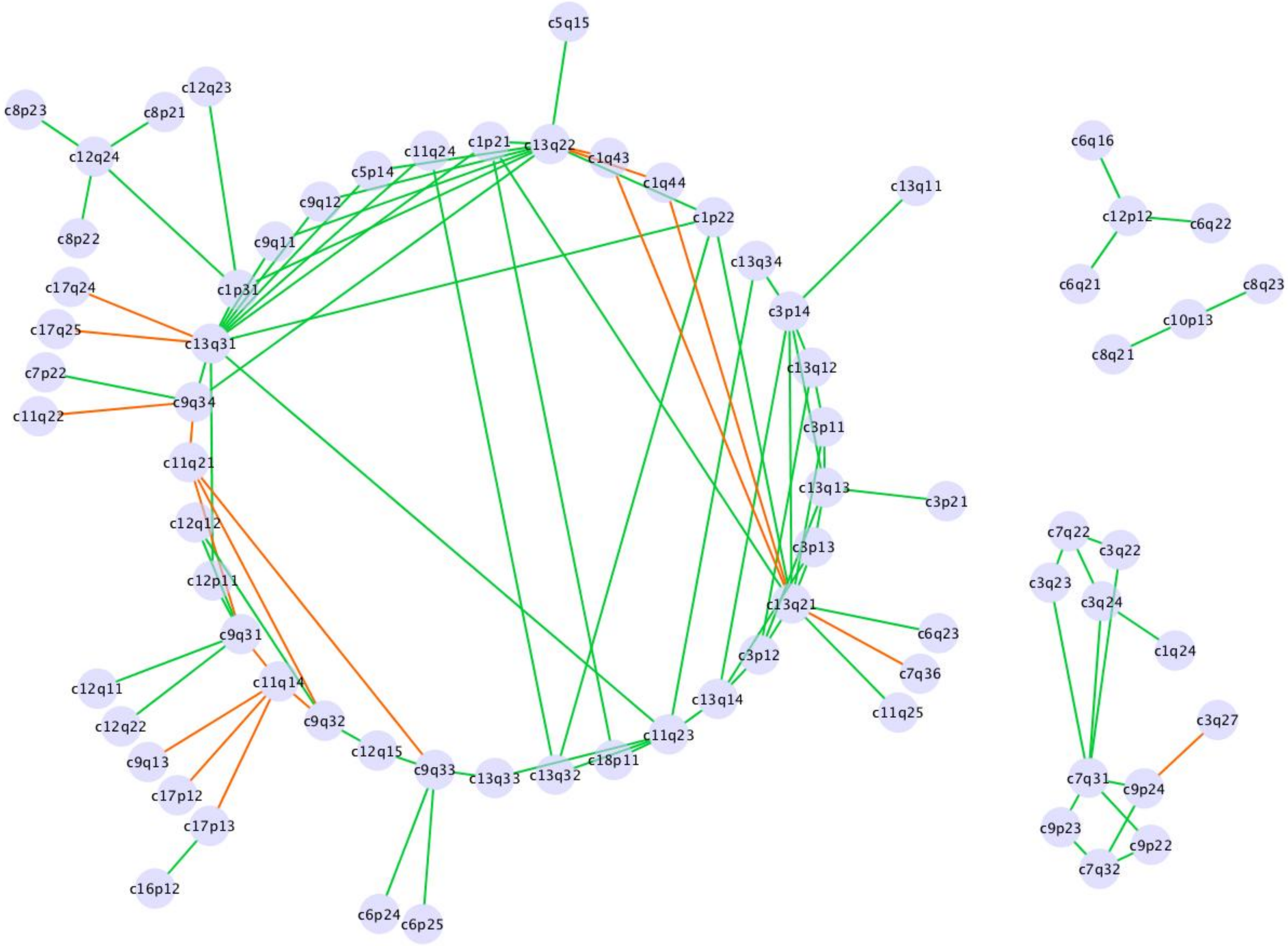
c5q15
c12q23
c8p23
c8p21
c12q24
c8p22
c11q24
c1p21
c13q22
c1q43
c1q44
c5p14
c9q12
c9q11
c1p22
c13q34
c13q11
c1p31
c17q24
c17q25
c13q31
c7p22
c9q34
c11q22
c11q21
c3p14
c13q12
c3p11
c13q13
c3p21
c12q12
c3p13
c12p11
c13q21
c6q23
c9q31
c3p12
c7q36
c11q14
c13q14
c11q25
c12q11
c9q32
c12q22
c11q23
c9q13
c12q15
c18p11
c9q33
c13q33
c13q32
c17p12
c17p13
c16p12
c6p24
c6p25
c6q16
c12p12
c6q22
c6q21
c8q23
c10p13
c8q21
c7q22
c3q22
c3q23
c3q24
c1q24
c3q27
c7q31
c9p24
c9p23
c9p22
c7q32
